# Cycle Threshold (Ct) Value Trends in COVID‐19: Analyzing Gender, Age, and Severity Factors Across Major Waves in India

**DOI:** 10.1002/iid3.70304

**Published:** 2026-02-25

**Authors:** Rudra Kumar Pandey, Vandana, Shailesh Desai, Prashanth Suravajhala, Gyaneshwer Chaubey

**Affiliations:** ^1^ Gyan Lab, Cytogenetics Unit, Department of Zoology Banaras Hindu University Varanasi India; ^2^ Department of Biosciences Manipal University Jaipur Jaipur Rajasthan India; ^3^ Bioclues.org, Hayatnagar Hyderabad India

**Keywords:** COVID‐19, Ct value, RT‐PCR, SARS‐CoV‐2, viral load

## Abstract

**Objectives:**

This study investigates the variations in cycle threshold (Ct) values from RT‐PCR tests across different COVID‐19 waves, age groups, and genders to assess their correlation with viral load, disease severity, and epidemiological indicators in India.

**Methods:**

We analysed a data set of 53,485 confirmed COVID‐19 cases, categorizing Ct values across three major COVID‐19 waves, age groups, and genders. Non‐parametric statistical tests were applied to compare Ct values, and linear regression analysis was conducted to evaluate the association between Ct values and COVID‐19 epidemiological indicators.

**Results:**

Ct values varied significantly across COVID‐19 waves, with the highest values in the first wave, the lowest in the second, and intermediate values in the third. Children exhibited the highest Ct values, while the elderly had the lowest. Females showed significantly lower Ct values than males. A negative correlation was observed between Ct values and COVID‐19 cases and deaths, indicating that lower Ct values reflected higher viral loads and more severe outbreaks.

**Conclusions:**

These findings highlight Ct values as a useful predictive marker for viral load and disease severity. Monitoring Ct values can enhance public health strategies by informing pandemic response efforts and resource allocation.

## Graphical abstract

(1) Analysis of 53,485 COVID‐19 cases show significant variations in Ct values over temporal, demographic and and epidemiological variables. (2) Ct values were highest in 1st wave, lowest in 2nd, and increased again in 3rd wave. (3) Ct values decrease with age with children shows highest and elderly lowest values. (4) Females show lower Ct than males suggesting potential hormones or immunity influence. (5) Ct values shows significant negative correlation with rising cases and deaths.

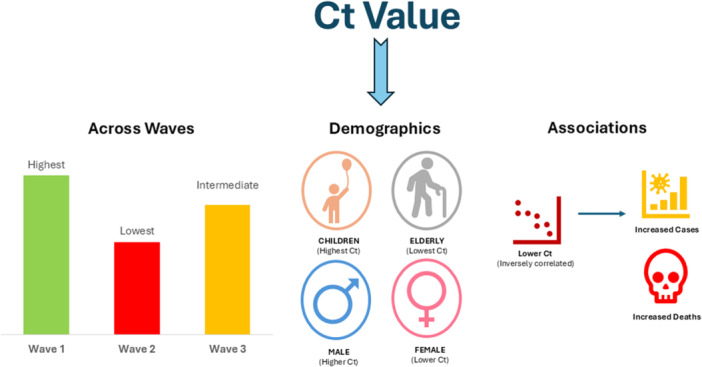

## 1 Introduction

The COVID‐19 pandemic, driven by the SARS‐CoV‐2 virus, has had a profound global impact, leading to unprecedented challenges in public health, socioeconomic structures, and healthcare systems [1]. Cases of unexplained pneumonia emerged in December 2019 in Wuhan, China. Respiratory, blood and faecal samples were collected, and deep sequencing analysis from the respiratory sample indicated the presence of a novel coronavirus, later named 2019‐nCoV [2]. Studies showed that the virus is a positive single‐stranded RNA virus belonging to the Coronaviridae family, with high homology to the coronavirus that caused SARS (Severe Acute Respiratory Syndrome) in 2002–2004 [3]. The World Health Organisation (WHO) officially named the disease COVID‐19, while the Coronavirus Study Group of the International Committee designated the virus SARS‐CoV‐2. COVID‐19 was declared the sixth public health emergency of international concern on January 30, 2020 [4].

The primary mode of SARS‐CoV‐2 transmission is through respiratory droplets and direct contact. Transmission is more likely in close physical proximity. However, respiratory droplets smaller than 100 micrometres in diameter can remain airborne for minutes and travel across rooms. Airborne transmission occurs when microorganisms within droplet nuclei linger in the air [5]. Various SARS‐CoV‐2 detection methods include RT‐PCR (reverse transcription polymerase chain reaction), serological testing, point‐of‐care diagnostics, nanotechnology‐based assays, and amplicon‐based metagenomic sequencing. Among these, RT‐PCR remains the gold standard due to its high sensitivity, requiring only a small RNA sample [6, 7, 8].

One of the critical factors in understanding the transmission dynamics and severity of COVID‐19 is the viral load, often measured through cycle threshold (Ct) values in RT‐PCR tests. Ct represents the number of cycles required for a fluorescent signal to become detectable, with an inverse relationship to viral load: lower Ct values [9, 10, 11, 12, 13, 14, 15, 16, 17, 18, 19] indicate a higher concentration of viral RNA, whereas higher Ct values imply lower viral loads. A Ct value below 40 is typically considered positive [20].

Ct values **can** play a significant role in monitoring COVID‐19 infection dynamics. Public health interventions, such as contact tracing and quarantine measures, can be tailored based on Ct values to mitigate viral spread. Additionally, Ct values serve as a crucial tool for evaluating the effectiveness of COVID‐19 vaccines. Despite extensive research, gaps remain in understanding the relationship between Ct values and various SARS‐CoV‐2 variants (e.g., Alpha, Delta, Omicron). It remains unclear whether observed Ct differences arise from intrinsic viral properties or external influences, such as host genetics, shifts in testing strategies or population immunity [21, 22, 23, 24, 25, 26, 27]. Furthermore, the association between Ct values and demographic factors (e.g., age, gender) and their correlation with epidemiological indicators remains inconsistent with heterogeneous difference in viral load [9, 10, 11, 12, 13, 14, 15, 16, 17, 18, 19, 28, 29]. Therefore, a comprehensive, large‐scale longitudinal multicentre study that examines variation in Ct values across these variables is limited and much needed.

This study utilises a comprehensive data‐set comprising 53,485 confirmed COVID‐19 cases in India to explore variations in Ct value over time across different pandemic waves, age groups, genders, SARS‐CoV‐2 variants, and epidemiological factors to provide insights into viral load progression and its correlation with disease severity and transmissibility. Such Longitudinal, multicenter studies that monitor Ct value dynamics will further clarify aspects of viral replication, shedding, and immune responses. This enhanced understanding will deepen our knowledge of the infectious period and the progression of the disease. By identifying key patterns and variations in Ct values, we aim to enhance the understanding of COVID‐19 transmission and inform public health strategies to optimise resource allocation and tailor interventions to mitigate the impact of future pandemics effectively.

## 2 Materials and Methods

### 2.1 Data Set

Data were obtained from the Indian Council of Medical Research (ICMR) via official data‐sharing agreements and involved no primary data collection. Before transfer, ICMR removed all personal identifiers and assigned a unique study identifier to each record. Use of the data set was limited to the analytical objectives described in the data‐sharing agreement. The data set includes 53,485 positive cases from various Indian states with patient demographics (age, gender), sample collection dates, and Ct values for E gene, RdRp, ORF1b. Epidemiological data were sourced from https://ourworldindata.org/ (Last accessed on February 1, 2025).

### 2.2 Inclusion/Exclusion Criteria

Records were eligible for inclusion if they represented RT‐PCR–confirmed SARS‐CoV‐2 infection in the ICMR data set, contained a numeric cycle‐threshold (Ct) value for at least one diagnostic target (E gene, RdRp, or ORF1b), reported a sample collection date within the study period, and included at least one essential covariate (age or sex). Records were excluded if the collection date were missing, if Ct values were reported as non‐numeric, undetermined, or outside the stated dynamic range of the originating assay, if the record was flagged by the reporting laboratory for quality control failure, or if the entry was a duplicate (defined as identical study identifier and collection date). Only the earliest positive sample (by collection date) was retained for the primary analysis for individuals with multiple positive specimens. For consistency and to maximise comparability across cases, Ct values from the E‐gene were used in the primary analysis, as this target was the most consistently reported across the data set and provided greater homogeneity for downstream comparisons.

### 2.3 Outlier Removal

Before each analysis, the outliers were identified and removed for each group to ensure the integrity of the data using the Tukey's Interquartile range (IQR) method. In the IQR method, Q1 (25th percentile) and Q3 (75th percentile) were calculated to find the IQR (IQR = Q1–Q3). The lower bound (Q1‐1.5*IQR) and upper bound (Q3 + 1.5*IQR) were calculated) and any data points that fall below or above the lower and upper bounds were identified as outliers and removed. Box‐and‐whiskers plots were used to display the distribution. The plot consists of a box representing the IQR, with a line inside marking the median. Whiskers, extend from the box to the minimum and maximum values and outliers are displayed as individual points beyond the whiskers.

### 2.4 Statistical Analysis

Normality was assessed using the Kolmogorov‐Smirnov and Shapiro‐Wilk tests. Due to deviations from normality, non‐parametric tests (Kruskal‐Wallis and Mann‐Whitney U) were employed. Further post‐hoc Dunn's tests with Bonferroni correction were used for pairwise comparisons. Linear regression analysis was used to examine associations between Ct values and epidemiological variables. All statistical analyses were performed using SPSS version 26, and composite graphics were generated with R. A significance threshold of *p* < 0.05 was applied for all tests.

## 3 Results

### 3.1 Comparison of Ct Values Across COVID‐19 Waves

**Figure 1:**
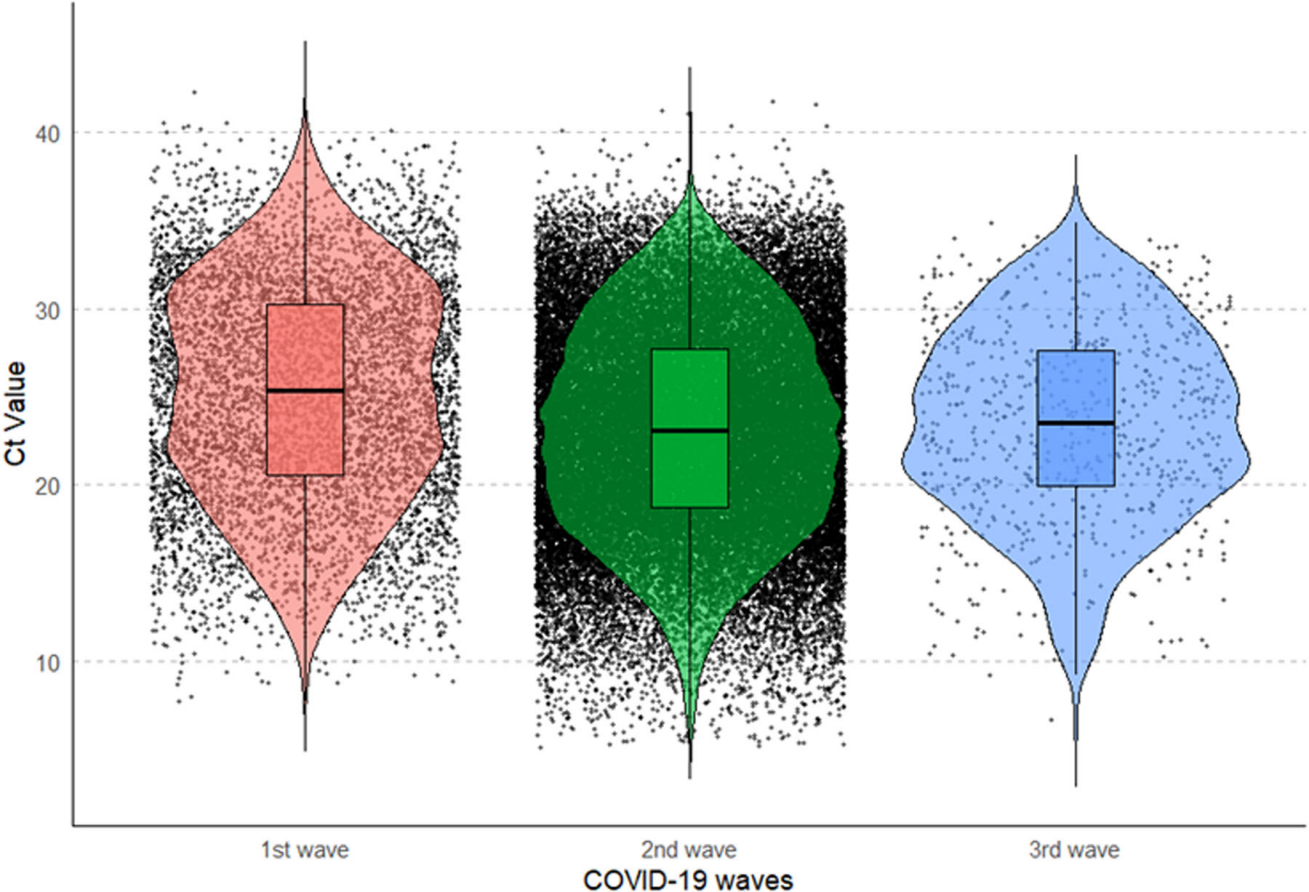
Violin plot showing the distribution of RT‐PCR Ct values during the three major COVID‐19 waves. The data highlights variations in Ct values across the different waves, with noticeable differences in medians and distribution shapes.

The Ct values were categorized by the three major COVID‐19 waves in India to compare the Ct values across these significant periods. (Supporting Information Table 1a). A Kruskal‐Wallis test revealed significant differences among three waves (*p* < 0.05) (Figure 1, Supporting Information Table 1b). Dunn's post‐hoc tests test for pairwise comparisons revealed significant difference in Ct values between the 1st and 2nd waves, as well as between the 1st and 3rd waves (adjusted *p* < 0.05, Supporting Information Table 1c). However, no significant variations in Ct values were observed between 2nd and 3rd wave. The first wave demonstrated the highest Ct values, indicating significantly lower viral loads during this period compared to subsequent waves. In contrast, the second wave recorded the lowest Ct values, representing the highest viral loads. The third wave presented Ct values that were intermediate i.e., higher than those in the second wave but lower than those of the first wave — indicating a moderate viral load during this course of time. Thus, Ct value was found to be highest in the 1st wave and lowest in the 2nd wave, and higher Ct value in the 3rd wave as compared to the 2nd wave (Supporting Information Table 1d).

To further understand the distribution of COVID‐19 cases by their Ct value, we divided Ct values into 3 categories as Low (< 25), intermediate (25–30) and High (> 30). In India, we observed the highest proportion of low Ct value cases (57.4%) followed by intermediate Ct value cases (29.2%) and the lowest proportion of High Ct value cases (13.4%) (Supporting Information Figure 2). We also analyzed the proportion of Ct value across 3 categories in each of the three COVID‐19 waves. We consistently observed the highest proportion of low Ct value cases followed by intermediate Ct value cases and the lowest proportion of High Ct value cases in each of the three COVID‐19 waves (Supporting Information Figure 3a–c). The 1st wave has the lowest proportion of low Ct value cases (46.1%), whereas the 2nd wave represents the highest proportion of low Ct value cases among the three waves (59.0%). The 3rd wave represents a decreased proportion of low Ct value (56.5%) as compared to 2nd wave, therefore conversely confirming our previous observation of the highest Ct value in the 1st wave and lowest in the 2nd wave, followed by a higher Ct value in the 3rd wave as compared to the 2nd wave (Figure 1).

### 3.2 Temporal Trends Within Each Wave

**Figure 2:**
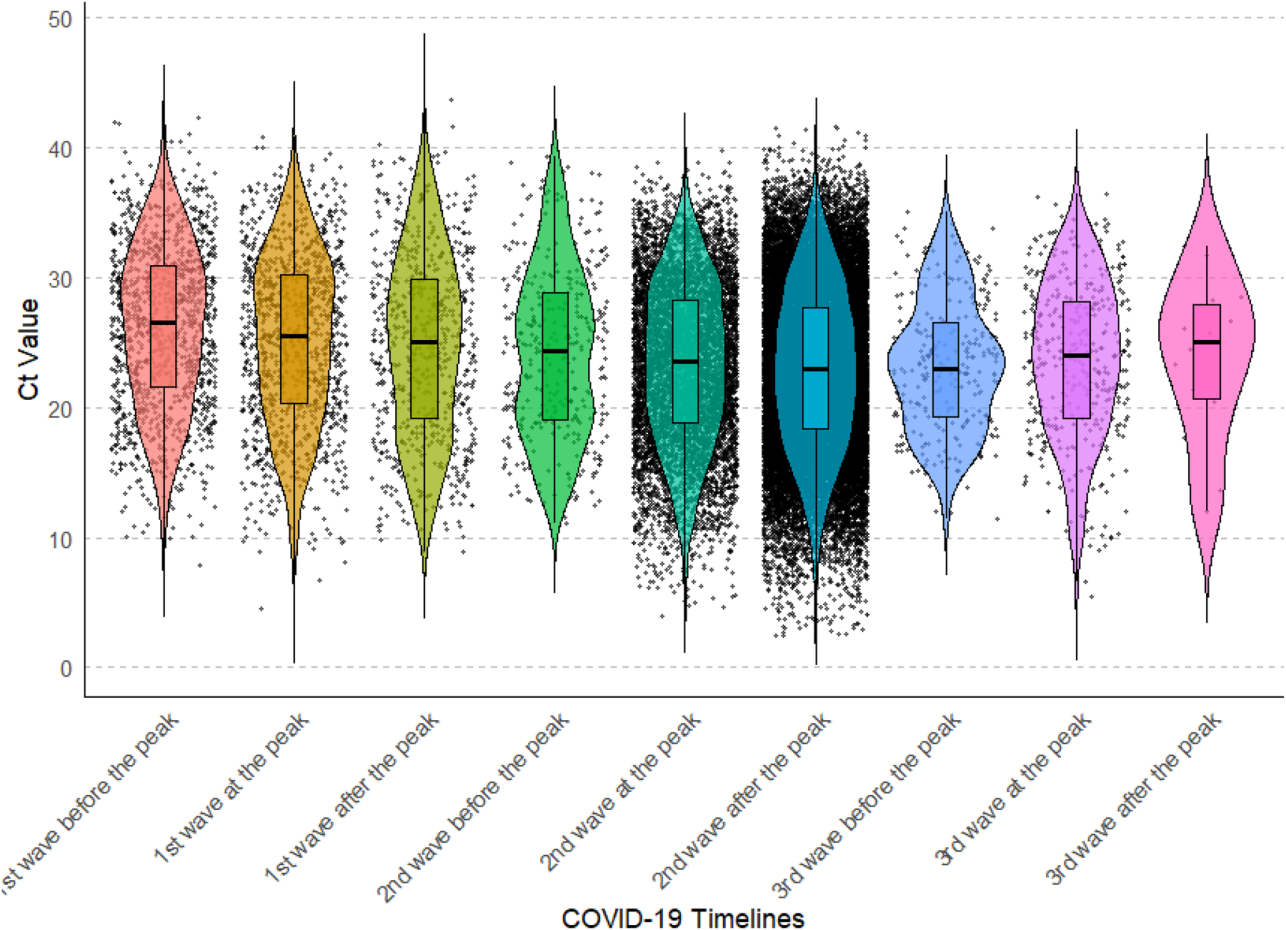
Violin plot showing the distribution of RT‐PCR Ct values across nine COVID‐19 timeline groups: before, at the peak, and after the peak of the 1st, 2nd, and 3rd waves. The data highlights trends in Ct values during different phases of the COVID‐19 waves with variations in the median and distribution shape among the groups.

To analyze the trend in Ct values across different phases of the three COVID‐19 Waves in India, the data was divided into 9 groups (Supporting Information Table 2a), with three groups corresponding to each wave: before, at and after the peak. Kruskal–Wallis test revealed a significant difference in Ct value across the nine groups (*p*‐value < 0.05, Figure 2 and Supporting Information Table 2b). In total 36 pairwise comparisons were made to identify significant differences among the groups; out of these, 15 groups showed significant differences across the various phases of each wave as well as between the waves (adjusted *p* < 0.05, Supporting Information Figure 4 and Supporting Information Table 2c). Notable differences were observed between phases of the 1st wave (e.g., before, at, and after the peak) and corresponding phases of the 2nd wave. The 2nd wave consistently exhibited lower Ct values, indicating higher viral loads compared to the 1st wave. However, no significant differences were found between the 2nd wave and the 3rd wave. Although the 3rd wave demonstrated higher Ct values, indicating lower viral loads, likely reflecting the impact of vaccination and acquired immunity. Overall, trend shows the highest median Ct values at the beginning of the pandemic, followed by a progressive decline that continued until the second wave, during which the lowest Ct values were recorded. After this point, a trend reversal occurred, with progressively higher Ct values observed throughout the third wave. (Supporting Information Table 2d).

### 3.3 Age‐Based Variations in Ct Values

**Figure 3:**
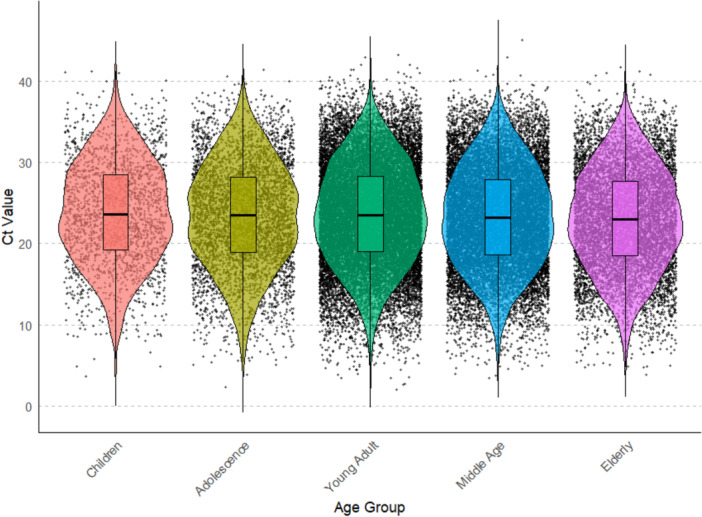
Violin plot showing the distribution of RT‐PCR Ct values across different age groups: Children, Adolescents, Young Adults, Middle Age, and Elderly. The plot highlights differences in Ct values across age groups, with variations in the median and distribution shape among the groups.

To investigate the Ct value in different age groups. The data‐set was stratified into five age groups: children (0–9), adolescents (10–19), young adults (20–39), middle‐aged (40–59), and elderly (≥ 60). A significant difference among these groups was found (*p* < 0.05) (Figure 3, Supporting Information Table 3a). A pairwise comparison was conducted for each group to identify groups with significant differences. In total, 10 comparisons were conducted for 5 groups; 5 groups were found to be significantly different from each other (adjusted *p* < 0.05, Supporting Information Figure 5a and Supporting Information Table 3b). Significant differences were observed in Ct values between Children vs Elderly, Adolescence vs Elderly, Young‐Adults vs Elderly, Children vs Middle‐Age and Young‐Adult ‐ Middle‐Age indicating variation in viral dynamics across age groups. The Ct value was observed to be highest in children and lowest in the elderly group (Supporting Information Table 3c). No significant differences were observed between groups like Children vs Young‐Adults, Adolescents versus Young‐Adults, Adolescents versus Children, Adolescents versus Middle‐Aged and Middle‐Aged vs Elderly. The lack of significant differences between adolescents and other groups suggests similar viral load levels for this age category. The overall trend shows that the Ct values generally decrease as age increases, with a slight increase in the Young Adult group.

We further analyze the Ct values across different age groups in each of the 3 COVID‐19 waves to investigate the impact of different COVID‐19 waves in various age groups. The data was divided into 3 COVID‐19 waves for each of the 5 age groups. The Kruskal Wallis test was done for each age group to compare differences in Ct values across 3 COVID‐19 waves. The test revealed a significant difference in Ct value across each age groups for 3 COVID‐19 waves (*p*‐value < 0.05, Supporting Information Figure 5b, Supporting Information Table 3d). Similar to the previous result, we found consistent observation of the highest Ct value in the 1st wave and lowest in the 2nd wave, followed by a higher Ct value in the 3rd wave as compared to the 2nd wave (Supporting Information Table 3e). With the three COVID‐19 waves, three pairwise comparisons were made for each age group, out of which 1st wave – 2nd wave and 1st wave – 3rd wave exhibited consistent significance in each age group (adjusted *p* < 0.05, Supporting Information Table 3f). Additionally, we examine the proportion of Ct values in three categories (low, intermediate and high) across the three COVID‐19 waves. Similar to the previous observation in each of the three COVID‐19 waves, we consistently found the largest proportion of low Ct value cases, followed by intermediate Ct value cases, and the lowest proportion of high Ct value cases (Supporting Information Figure 5c).

### 3.4 Gender Differences in Ct Values

**Figure 4:**
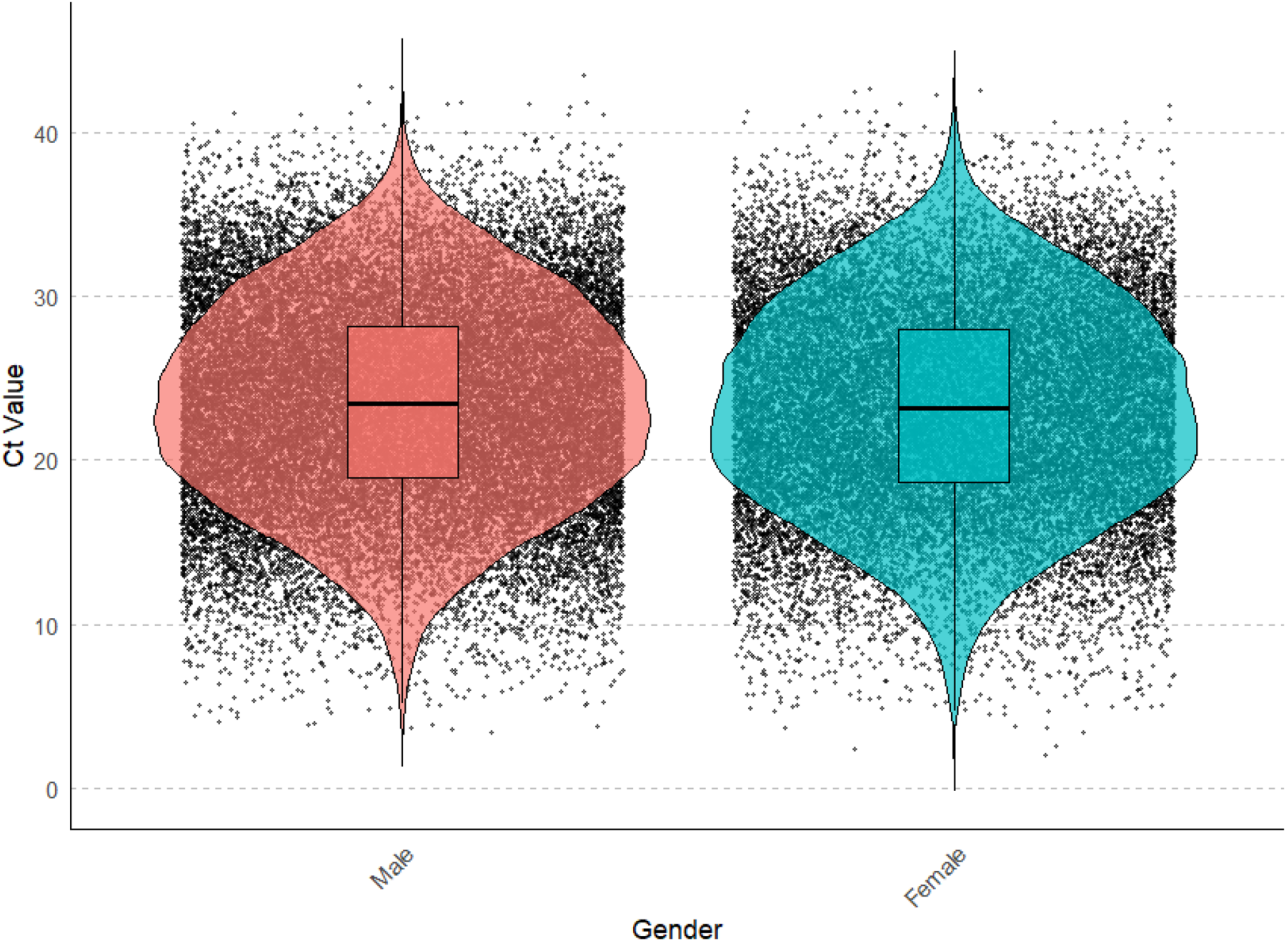
Violin plot showing the distribution of Ct values for COVID‐19 RT‐PCR tests stratified by gender. The left panel represents Male participants (pink), and the right panel represents female participants (blue).

To analyze the impact of gender on Ct values, data was divided into two groups: male and female. The Mann‐Whitney U test was performed to compare the difference in Ct values between the two genders. The test revealed a significant difference between 2 groups (*p*‐value < 0.05). The higher mean rank for males (26,997.94 vs. 26,331.25) suggests that male Ct values are, on average, higher than female Ct values, suggesting lower viral loads in males than females (Figure 4, Supporting Information Table 4a,b).

As in previous observations, we observed the highest proportion of low Ct value followed by intermediate Ct value and the lowest proportion of High Ct value in both genders (Supporting Information Figure 6a). We also investigated the Ct value in both genders across 3 waves for this Kruskal–Wallis test was performed. The test revealed significant differences for both genders across 3 waves (Supporting Information Figure 6b, Supplementary Table 4c). Consistent as per previous observation, the Ct value was found to be highest in the 1st wave and lowest in the 2nd wave, followed by a higher Ct value in the 3rd wave as compared to the 2nd wave (Supporting Information Table 4d). With the three COVID‐19 waves, three pairwise comparisons were made to identify groups that differ significantly. Similar to the previous observation, significant differences (adjusted *p* < 0.05) were observed between 1st wave – 2nd wave and 1st wave – 3rd wave in both genders (Supporting Information Table 4e).

### 3.5 Association With Epidemiological Variables

**Figure 5:**
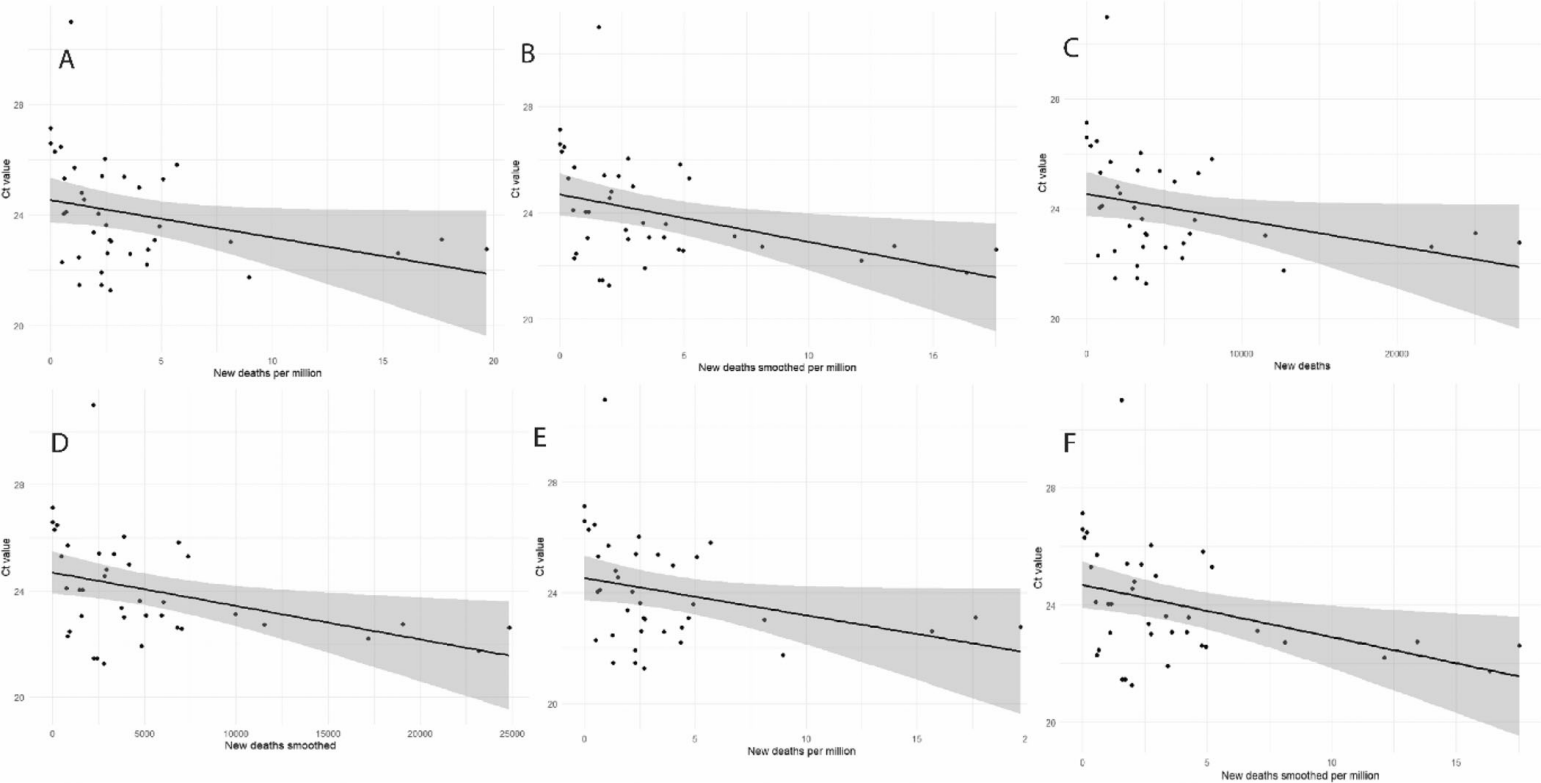
Linear regression analysis depicting the relationship between COVID‐19 RT‐PCR Ct values and various epidemiological indicators, including new cases (smoothed), new cases per million, new deaths, new deaths per million, new deaths smoothed, and new deaths smoothed per million (A, B, C, D, E and F respectively). Scatter plots represent individual data points, while fitted regression lines shows the fitted regression model for each indicator. Shaded areas around the lines represent 95% confidence intervals. All indicators show a significant negative slope.

To understand how changes in the Ct value might relate to variations in COVID‐19 epidemiological variables, linear regression analysis was performed to test the epidemiological association, if any. For this, the average of Ct values of 15‐day intervals was computed across all three waves of the COVID‐19 pandemic in India with corresponding COVID‐19 epidemiological variables. The analysis reveals a consistently significant negative correlation (*p*‐value < 0.05) between the Ct value and various indicators of COVID‐19 spread and severity. Specifically, as the Ct value decreases, there is an increase in the new cases (smoothed), new cases per million (smoothed), new deaths, new deaths (smoothed), new deaths per million, and new deaths per million (smoothed) (Figure 5a–f and Supporting Information Table 5).

## 4 Discussion

India was dominated by three major COVID‐19 waves (Supporting Information Figure 1). Each wave presents itself with varying levels of transmissibility and severity [30, 31, 32, 33]. Ct values from RT–PCR are commonly used as a semi‐quantitative proxy for viral RNA load therefore the comparison of Ct values across COVID‐19 waves could reveal insights into the viral load dynamics over time. Our analysis observed significant differences in Ct values across three COVID‐19 Waves (Figure 1 and Supporting Information Table 1b,c). The Ct value was observed to be highest in the 1st wave, suggesting that the viral loads were lower in the initial phase of the pandemic, which could be attributed to the less transmissible Wild type variant, strict lockdown, quarantine and social distancing policies implemented during the 1st wave. The 2nd wave, characterized by the lowest Ct values, indicates a higher viral load, associating with a more transmissible or severe Delta variant predominant during this period and more relaxed policies. The 3rd wave showed an increase in Ct values compared to the 2nd wave; the intermediate Ct values suggest a decrease in viral load compared to the 2nd wave (Supporting Information Table 1d), which could be attributed to the milder Omicron variant prevalent during this period and the massive vaccination drive in India after the second wave [34, 35, 36] and partially immune population from the two previous waves leading to less severe infections despite ongoing transmission, however this difference was not significant suggesting RT‐PCR Ct values appear to remain largely unaffected by an individual's vaccination status [37, 38, 39].

To analyze the Ct values trend across different phases of each of the three COVID‐19 waves in India, the data was segmented into 9 groups. These groups represent three phases for each wave, i.e., before the peak, at the peak, and after the peak of the wave (Supporting Information Figure 1 and Supporting Information Table 2a). Our analysis indicated a significant difference in Ct values across these nine groups (Supporting Information Table 2b), both within and between different waves (Figure 2, Supporting Information Figure 4 and Supporting Information Table 2c) Ct value was high at the beginning of the 1st wave, possibly due to lower virus load, less transmissible variants like Alpha, social distancing, rigorous lockdowns and isolation measures deployed during the early stages of the pandemic. Thereafter Ct value decreases progressively up to the 2nd wave which could be explained by higher viral load, contagious variants like Delta and lenient policies during this time in India. After the peak of the 2nd wave, Ct value started to increase progressively, reaching the highest value observed after the peak of the 3rd wave (Supporting Information Table 2d), which suggests a decrease in viral loads as the wave subsides, possibly due to milder omicron variant, increased immunity from the previous waves and massive vaccination drive and effective interventions [38, 40].

The COVID‐19 pandemic has presented varying impacts across different age groups. Previous observational studies across different age groups have produced heterogeneous results with no consistent difference in viral load [9, 10, 11, 12, 13, 14, 15, 16, 17, 18, 19, 28, 29]. Given this heterogeneity, we included age as a covariate and interpret any age–Ct relationships. For this purpose, Ct values were divided into 5 different age groups. Our analysis revealed significant differences in Ct values across the five distinct age groups (Figure 3, Supporting Information Figure 5a and Supporting Information Table 3a,b). The highest Ct values were observed in children, while the lowest were found in the elderly group. The overall trend indicates a general decrease in Ct values with increasing age, with a notable exception in the young adult group (20–39 years), which exhibited a slight increase (Supporting Information Table 3c). The observation of highest Ct values in children and lowest in the elderly suggests distinct differences in viral loads across age groups. Higher Ct values in children indicate lower viral loads, likely due to stronger innate immune responses, reduced transmission dynamics, and milder or asymptomatic infections. In contrast, the lowest Ct values in the elderly reflect higher viral loads, which can be attributed to higher risk, weakened immune responses and increased disease severity,suggesting advanced age is a strong predictor of severe COVID‐19 due to immunosenescence and comorbidity burden, and could therefore influence viral dynamics and Ct measurements [41]. Our further analysis of Ct values across the three COVID‐19 waves for each age group demonstrated significant differences within each group. Consistent with the initial findings, Ct values varied notably across the waves. The first wave generally exhibited the highest Ct values, followed by a decrease in the second wave and a subsequent increase in the third wave (Supporting Information Figure 5b). This pattern held true across all age groups in each of the three waves except for children, who showed no significant differences in Ct values across the three waves (Supporting Information Table 3d), which may indicate a unique response to the virus or differences in testing and diagnosis protocols for this age group.

Sex differences in immune responses are well documented and likely arise from hormonal and genetic differences that modulate innate and adaptive immunity [42, 43]. Because these differences can influence viral replication kinetics and clinical outcomes, sex (male vs. female) is a biologically plausible covariate when examining Ct values. Empirical studies of SARS‐CoV‐2 have reported mixed results [11, 12, 13, 15, 44, 45] and therefore need further examination on large and diverse datasets. Therefore, we further investigate the impact of gender on cycle Ct values in COVID‐19 patients and analyze the variations in Ct values across three waves of the pandemic. Our analysis revealed a statistically significant difference in Ct values between males and females, with females exhibiting lower Ct values compared to males (Figure 4 and Supporting Information Table 4a,b), indicating a higher viral load in females. Biological factors, such as hormonal differences and immune response variations, might contribute to these observed disparities [46]. However, further research is necessary to elucidate the underlying mechanisms driving this gender difference in Ct values. Further, analysis of Ct value s across the three waves of the COVID‐19 pandemic also revealed significant differences for both genders. Consistent with previous observations, the Ct value was highest during the first wave, lowest during the second wave, and then increased again in the third wave. This trend suggests that the viral load in patients varied significantly in both genders across different phases of the pandemic (Supporting Information Figure 6b and Supporting Information Table 4c–e). The highest Ct values in the first wave might be attributed to the initial spread of the virus when the population had not yet developed widespread immunity, leading to higher transmission rates, while lower Ct values in 2nd wave indicate higher viral load and high transmissible virus strain like delta during the 2nd wave, followed by an increase in Ct value in 3rd wave as compared to 2nd wave indicate the role of herd immunity acquired from previous wave and massive vaccination drive after the second wave [47].

We also look for the proportion of cases in each of the three waves by Ct value to observe what proportion of cases fell into one of the three Ct value categories, i.e., Low (< 25), Intermediate [17, 18, 19, 28, 29, 30], and High (> 30). Across all three waves, the highest proportion of cases fell into the Low Ct value category, followed by Intermediate and then High Ct value categories (Supporting Information Figure 3a–c). We find similar observations in age and gender groups (Supporting Information Figures 5c and 6a). The consistent finding of the highest proportion of low Ct values across all waves suggests that low Ct values and high viral loads dominated the majority of COVID‐19 cases in India.

Finally, we investigated the relationship between Ct values and various COVID‐19 epidemiological variables to understand how variations in Ct values reflect changes in viral load, infection rates and mortality. Our analysis reveals a consistently significant negative correlation between the Ct value and various COVID‐19 epidemiological variables, such as cases and deaths across the three waves of the COVID‐19 pandemic in India (Figure 5a–f and Supporting Information Table 5). This relationship suggests that lower Ct values trend are associated with higher cases and severe epidemiological outcomes.

## 5 Conclusion

This study provides a comprehensive analysis of Ct value dynamics across three major COVID‐19 waves in India. The findings highlight that viral load varied substantially over time, with the highest Ct values (lowest viral loads) observed in the early phase, a marked decrease during the Delta‐driven second wave, and a moderate recovery in the Omicron‐dominated third wave. Further, this study highlights that age and gender significantly influence Ct values, with children showing higher Ct values than the elderly, and females displaying lower Ct values than males. The significant inverse correlation between Ct values and epidemiological outcomes of transmission and mortality supports the role of Ct monitoring as a predictive tool in managing COVID‐19 and therefore, can be used to inform tailored public health strategies, resource allocation, and future pandemic preparedness.

## Author Contributions

**Rudra Kumar Pandey:** data curation, formal analysis, investigation, methodology, resources, software, supervision, validation, visualization, writing – original draft, writing – review and editing. **Vandana:** data curation, formal analysis, visualization. **Shailesh Desai:** data curation, resources, validation, writing – review and editing. **Prashanth Suravajhala:** validation, writing – review and editing. **Gyaneshwer Chaubey:** conceptualization, funding acquisition, investigation, methodology, project administration, resources, supervision, validation, writing – review and editing.

## Ethics Statement

The data was obtained from the Indian Council of Medical Research (ICMR) through official cooperation and data‐sharing agreements with the relevant authorities and involved no primary data collection, therefore ethical approval was not needed.

## Conflicts of Interest

The authors declare that they have no known competing financial interests or personal relationships that could have appeared to influence the work reported in this paper.

## Declaration of and AI‐Assisted Technologies in the Writing Process

During the preparation of this study the author(s) used ChatGPT to enhance writing. After using this tool/service, the author(s) reviewed and edited the content as needed and take(s) full responsibility for the content of the publication.

## Supporting information


**Supp. Fig. 1:** Trends in daily new confirmed COVID‐19 cases (A) and SARS‐CoV‐2 variants prevalence (B) in India, illustrating multiple infection waves and the shifting dominance of variants over time. **Supp. Fig. 2:** Pie chart depicting overall distribution of COVID‐19 cases in India based on RT‐PCR Ct values categories as Low (30). **Supp. Fig. 3:** Pie chart depicting distribution of COVID‐19 cases in 1st, 2nd and 3rd waves respectively (a, b and c) based on RT‐PCR Ct values categories as Low (<25), intermediate (25‐30) and High (>30). **Supp. Fig. 4:** The heatmap represents the results of pairwise statistical comparisons between different phases of the 1st, 2nd, and 3rd COVID‐19 waves in India as shown on the x‐axis and y‐axis. **Supp. Fig. 5a:** The heatmap represents the results of pairwise statistical comparisons between different Age groups as shown on the x‐axis and y‐axis. **Supp. Fig. 5b:** Violin plot depicting the distribution of Ct values for COVID‐19 RT‐PCR tests across different age groups (Children, Adolescence, Young Adult, Middle Age, and Elderly). **Supp. Fig 5c:** Pie chart depicting distribution of COVID‐19 cases in Children, Adolescence, Young Adults, Middle Age and Elderly respectively (a, b, c, d and e) based on RT‐PCR Ct values categories as Low (30). **Supp. Fig. 6a:** Pie chart depicting distribution of COVID‐19 cases in Males and Females respectively (a and b) based on RT‐PCR Ct values categories as Low (30). **Supp. Fig. 6b:** Violin plots show the density distribution of Ct values for males and females during the 1st wave (red), 2nd wave (yellow), and 3rd wave (blue).

**Supp. Tab. 1a:** Timeline of the three major COVID‐19 waves used, showing their start and end months. **Supp. Tab. 1b:** The table presents the results of the independent‐samples Kruskal‐Wallis Test, used to compare the distributions of Ct Values across 3 waves. **Supp. Tab. 1c:** Dunn's post hoc pairwise comparison test across 3 waves. **Supp. Tab. 1d:** The table summarizes the mean and median COVID‐19 RT‐PCR Ct values across 3 COVID‐19 waves. **Supp. Tab. 2a:** Detailed timeline of each COVID‐19 wave used, highlighting pre‐peak, peak, and post‐peak periods. **Supp. Tab. 2b:** The table presents the results of the independent‐samples Kruskal‐Wallis Test, used to compare the distributions of Ct Values across different timelines. **Supp. Tab. 2c:** Dunn's post hoc pairwise comparison test across different timeline. **Supp. Tab. 2d:** The table summarizes the mean and median COVID‐19 RT‐PCR Ct values across different timelines. **Supp. Tab. 3a:** The table presents the results of the independent‐samples Kruskal‐Wallis Test, used to compare the distributions of Ct Values across different age groups. **Supp. Tab. 3b:** Dunn's post hoc pairwise comparison test across different Age Groups. **Supp. Tab. 3c:** The table summarizes the mean and median COVID‐19 RT‐PCR Ct values across different Age groups. **Supp. Tab. 3d:** The table presents the results of the independent‐samples Kruskal‐Wallis Test, used to compare the distributions of Ct Values in different age groups across 3 waves. **Supp. Tab. 3e:** The table summarizes the mean and median COVID‐19 RT‐PCR Ct values across different Age groups across 3 waves. **Supp. Tab. 3e:** Dunn's post hoc pairwise comparison test in different age groups across 3 waves. **Supp. Tab. 4a:** The table presents the results of the independent‐samples Mann‐Whitney U Test, used to compare the distributions of Ct Values in Males and Females. **Supp. Tab. 4b:** The table summarizes the mean and median COVID‐19 RT‐PCR Ct values in Males and Females. **Supp. Tab. 4c:** The table presents the results of the Independent‐Samples Kruskal‐Wallis Test, used to compare the distributions of Ct Values in Males and Females across 3 waves. **Supp. Tab. 4d:** The table summarizes the mean and median COVID‐19 RT‐PCR Ct values in Males and Females across 3 waves. **Supp. Tab. 4e:** Dunn's post hoc pairwise comparison test in Males and Female across 3 waves. **Supp. Tab. 5:** Linear regression analysis depicting the relationship between COVID‐19 RT‐PCR Ct values and various epidemiological indicators.

## Data Availability

All datasets generated for this study are included in the article/Supporting Material.
