# Supplementary material for: Cycle Threshold (Ct) Value Trends in COVID‐19: Analyzing Gender, Age, and Severity Factors Across Major Waves in India
Source: Immun Inflamm Dis. 2026 Feb 25;14(2):e70304. doi: 10.1002/iid3.70304 (PMC12933256; doi:10.1002/iid3.70304)
Supplement: Supplementary file 1 — Supp. Fig. 1: Trends in daily new confirmed COVID‐19 cases (A) and SARS‐CoV‐2 variants prevalence (B) in India, illustrating multiple infection waves and the shifting dominance of variants over time. Supp. Fig. 2: Pie chart depicting overall distribution of COVID‐19 cases in India based on RT‐PCR Ct values categories as Low (30). Supp. Fig. 3: Pie chart depicting distribution of COVID‐19 cases in 1st, 2nd and 3rd waves respectively (a, b and c) based on RT‐PCR Ct values categories as Low (<25), intermediate (25‐30) and High (>30). Supp. Fig. 4: The heatmap represents the results of pairwise statistical comparisons between different phases of the 1st, 2nd, and 3rd COVID‐19 waves in India as shown on the x‐axis and y‐axis. Supp. Fig. 5a: The heatmap represents the results of pairwise statistical comparisons between different Age groups as shown on the x‐axis and y‐axis. Supp. Fig. 5b: Violin plot depicting the distribution of Ct values for COVID‐19 RT‐PCR tests across different age groups (Children, Adolescence, Young Adult, Middle Age, and Elderly). Supp. Fig 5c: Pie chart depicting distribution of COVID‐19 cases in Children, Adolescence, Young Adults, Middle Age and Elderly respectively (a, b, c, d and e) based on RT‐PCR Ct values categories as Low (30). Supp. Fig. 6a: Pie chart depicting distribution of COVID‐19 cases in Males and Females respectively (a and b) based on RT‐PCR Ct values categories as Low (30). Supp. Fig. 6b: Violin plots show the density distribution of Ct values for males and females during the 1st wave (red), 2nd wave (yellow), and 3rd wave (blue). [file IID3-14-e70304-s002.docx]

**Supplementary Figures**


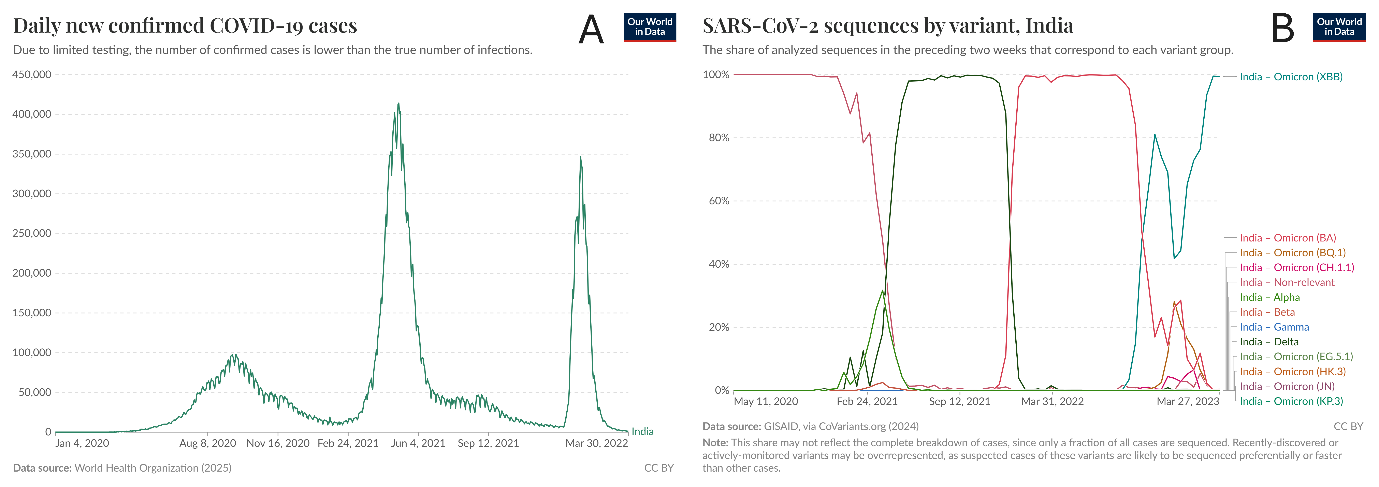


**Supp. Fig. 1** Trends in daily new confirmed COVID-19 cases (A) and SARS-CoV-2 variants prevalence (B) in India, illustrating multiple infection waves and the shifting dominance of variants over time.


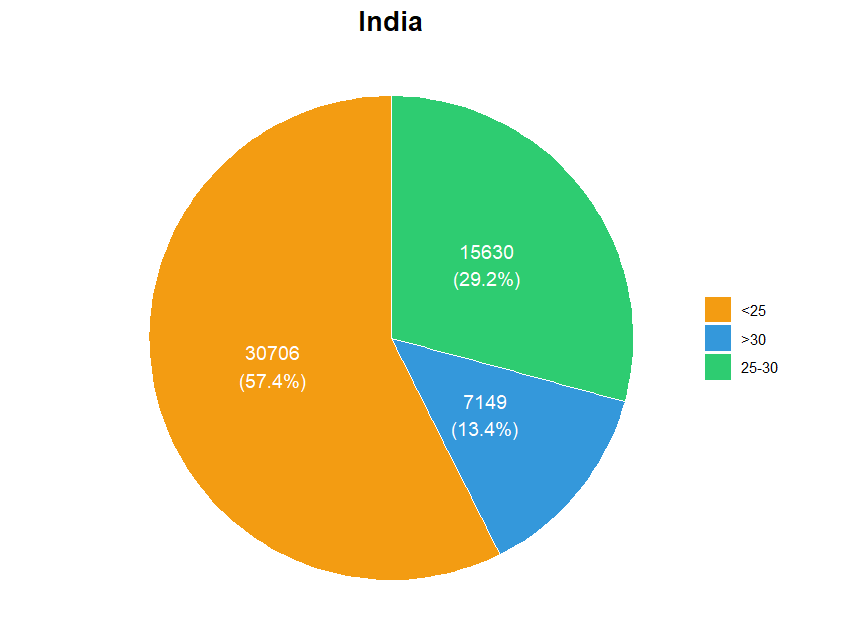


**Supp. Fig. 2.** Pie chart depicting overall distribution of COVID-19 cases in India based on RT-PCR Ct values categories as Low (<25), intermediate (25-30) and High (>30).


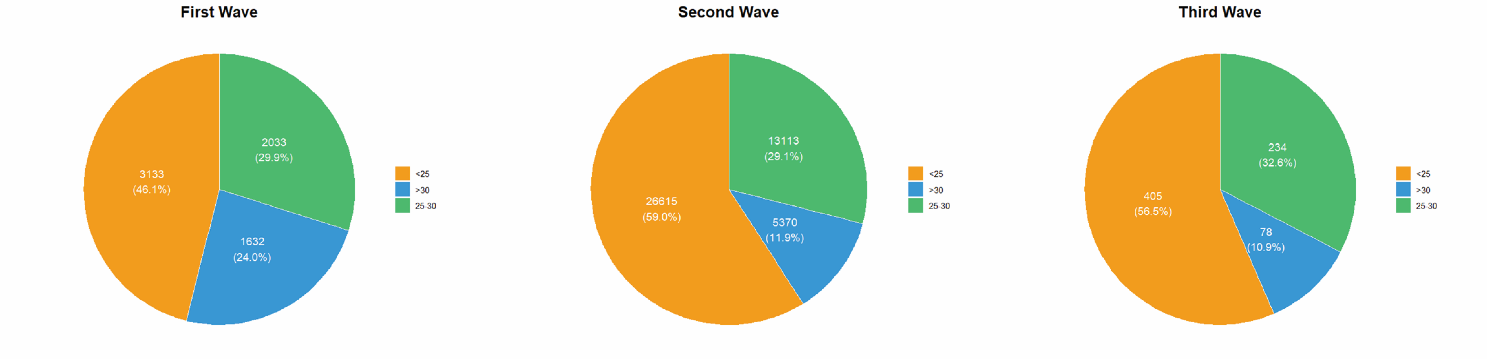
**Supp. Fig. 3.** Pie chart depicting distribution of COVID-19 cases in 1^st^, 2^nd^ and 3^rd^ waves respectively (a, b and c) based on RT-PCR Ct values categories as Low (<25), intermediate (25-30) and High (>30).


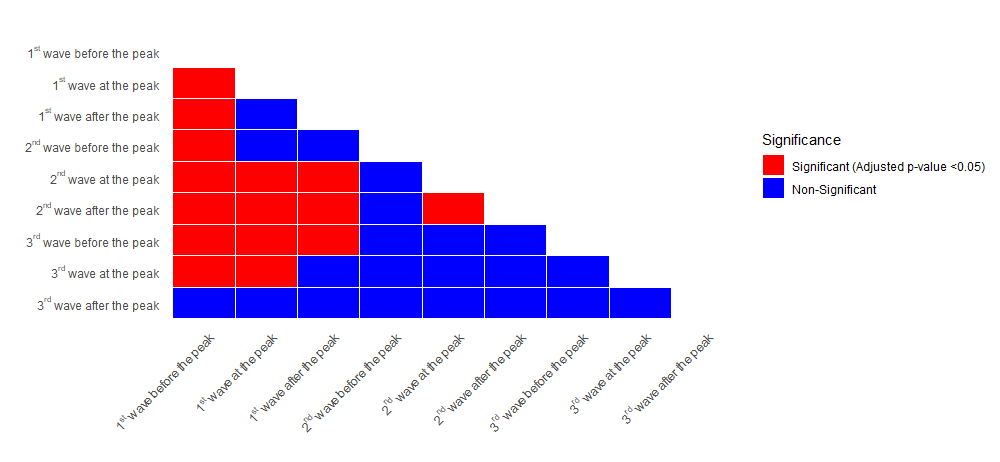


**Supp. Fig. 4.** The heatmap represents the results of pairwise statistical comparisons between different phases of the 1st, 2nd, and 3rd COVID-19 waves in India as shown on the x-axis and y-axis.


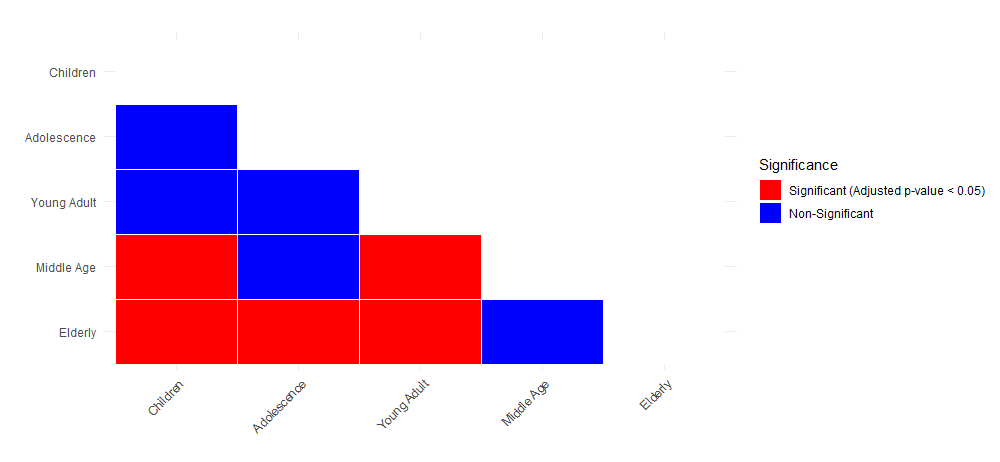


**Figure 5a.** The heatmap represents the results of pairwise statistical comparisons between different Age groups as shown on the x-axis and y-axis.


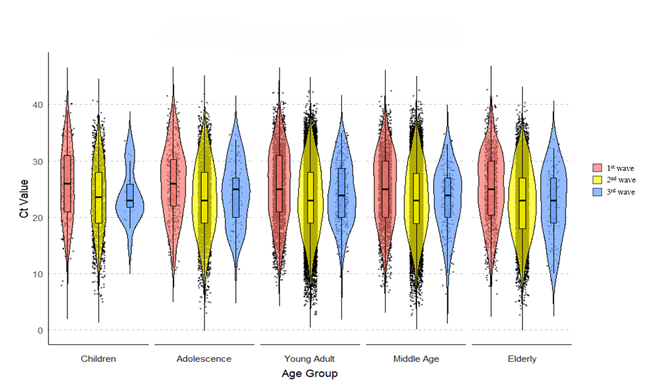


**Supp. Fig. 5b.** Violin plot depicting the distribution of Ct values for COVID-19 RT-PCR tests across different age groups (Children, Adolescence, Young Adult, Middle Age, and Elderly). Each age group is further stratified by pandemic waves: the 1st wave (pink), 2nd wave (yellow), and 3rd wave (blue). The plot highlights differences in Ct values in each age group across 3 waves, with variations in the median and distribution shape among the groups.


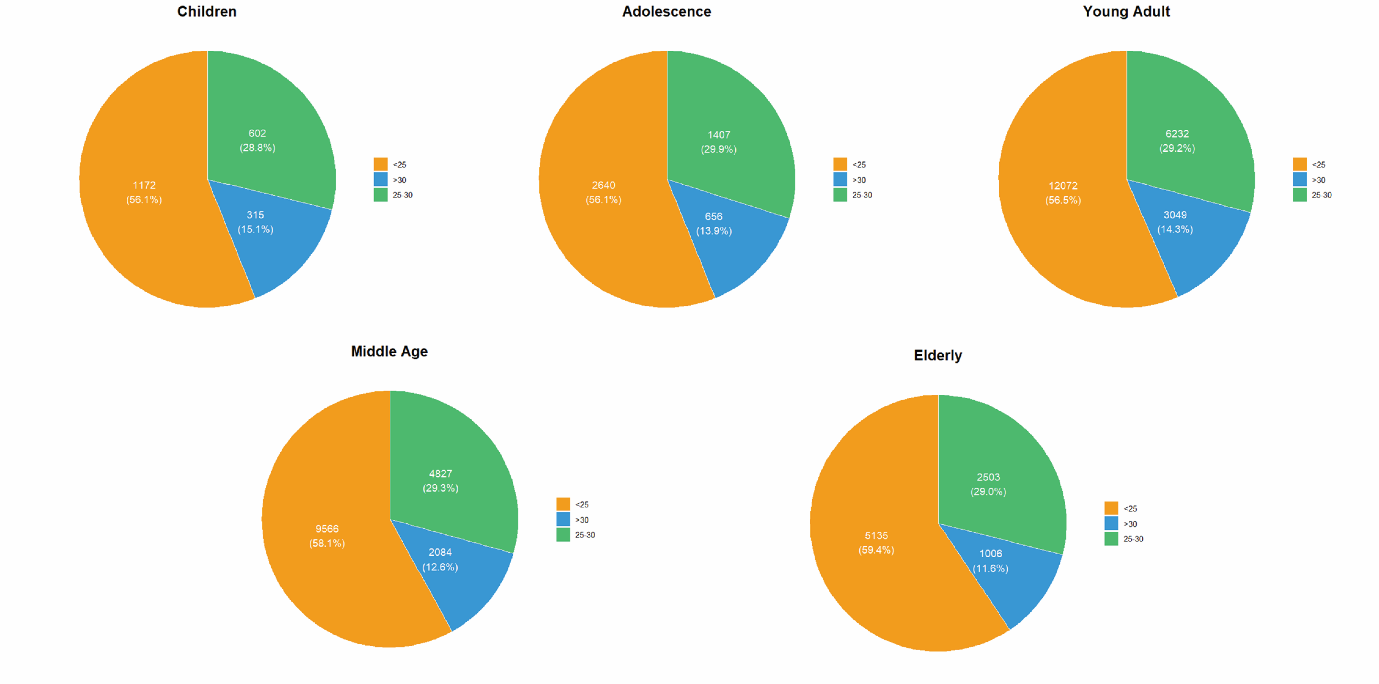


**Supp. Fig 5c.** Pie chart depicting distribution of COVID-19 cases in Children, Adolescence, Young Adults, Middle Age and Elderly respectively (a, b, c, d and e) based on RT-PCR Ct values categories as Low (<25), intermediate (25-30) and High (>30).


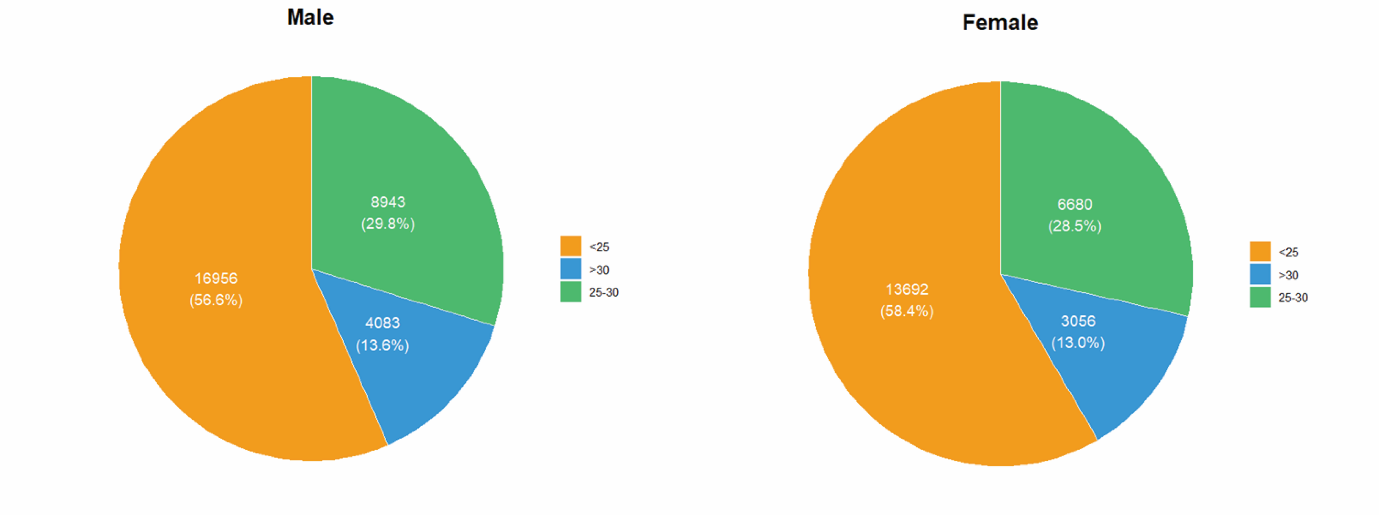


**Supp. Fig. 6a.** Pie chart depicting distribution of COVID-19 cases in Males and Females respectively (a and b) based on RT-PCR Ct values categories as Low (<25), intermediate (25-30) and High (>30).


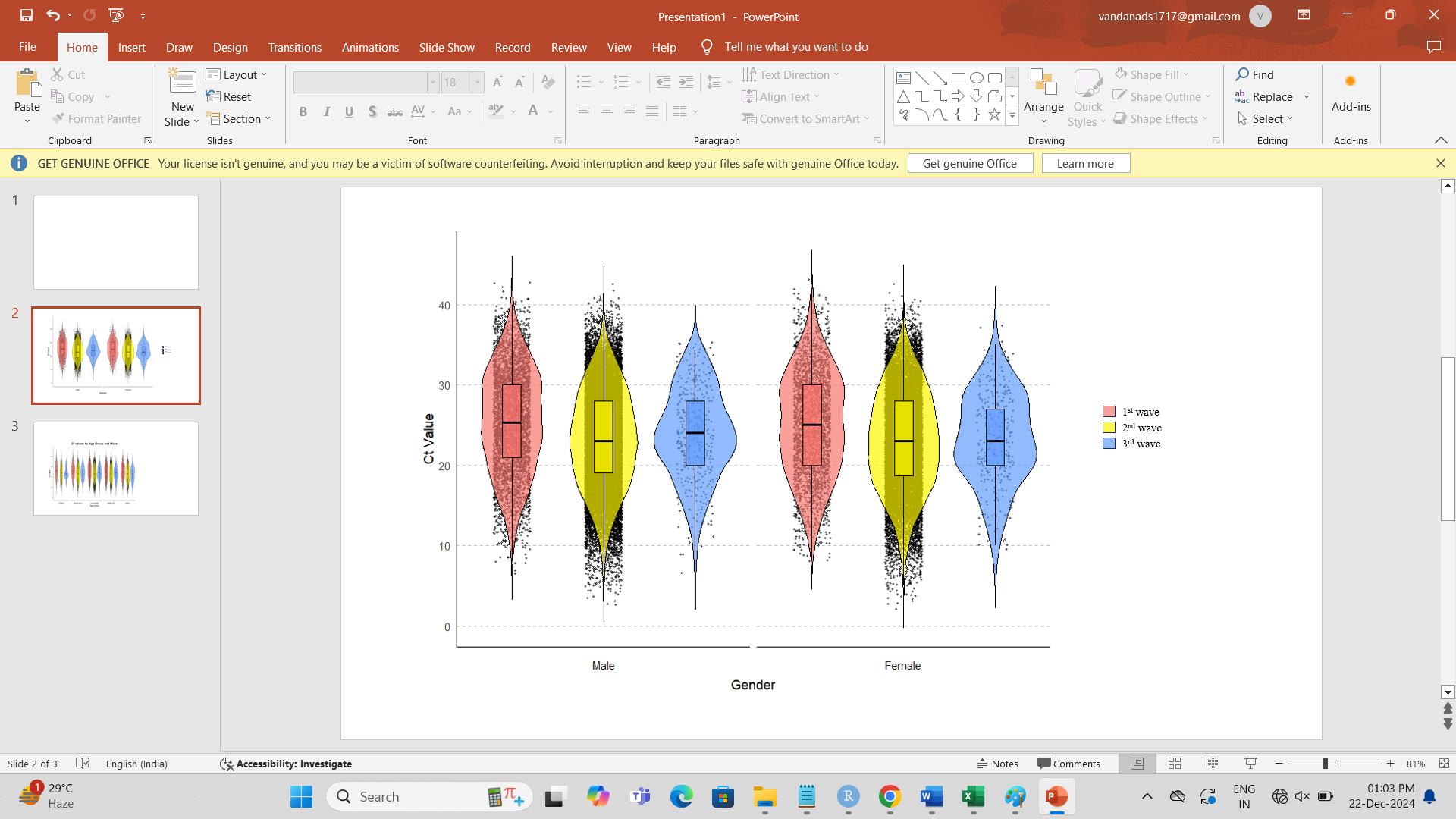


**Supp. Fig. 6b.** Violin plots show the density distribution of Ct values for males and females during the 1st wave (red), 2nd wave (yellow), and 3rd wave (blue). The plot highlights differences in Ct values for males and females across 3 waves, with variations in the median and distribution shape among the groups.
