# Supplementary material for: Cycle Threshold (Ct) Value Trends in COVID‐19: Analyzing Gender, Age, and Severity Factors Across Major Waves in India
Source: Immun Inflamm Dis. 2026 Feb 25;14(2):e70304. doi: 10.1002/iid3.70304 (PMC12933256; doi:10.1002/iid3.70304)
Supplement: Supplementary file 2 — Supp. Tab. 1a: Timeline of the three major COVID‐19 waves used, showing their start and end months. Supp. Tab. 1b: The table presents the results of the independent‐samples Kruskal‐Wallis Test, used to compare the distributions of Ct Values across 3 waves. Supp. Tab. 1c: Dunn's post hoc pairwise comparison test across 3 waves. Supp. Tab. 1d: The table summarizes the mean and median COVID‐19 RT‐PCR Ct values across 3 COVID‐19 waves. Supp. Tab. 2a: Detailed timeline of each COVID‐19 wave used, highlighting pre‐peak, peak, and post‐peak periods. Supp. Tab. 2b: The table presents the results of the independent‐samples Kruskal‐Wallis Test, used to compare the distributions of Ct Values across different timelines. Supp. Tab. 2c: Dunn's post hoc pairwise comparison test across different timeline. Supp. Tab. 2d: The table summarizes the mean and median COVID‐19 RT‐PCR Ct values across different timelines. Supp. Tab. 3a: The table presents the results of the independent‐samples Kruskal‐Wallis Test, used to compare the distributions of Ct Values across different age groups. Supp. Tab. 3b: Dunn's post hoc pairwise comparison test across different Age Groups. Supp. Tab. 3c: The table summarizes the mean and median COVID‐19 RT‐PCR Ct values across different Age groups. Supp. Tab. 3d: The table presents the results of the independent‐samples Kruskal‐Wallis Test, used to compare the distributions of Ct Values in different age groups across 3 waves. Supp. Tab. 3e: The table summarizes the mean and median COVID‐19 RT‐PCR Ct values across different Age groups across 3 waves. Supp. Tab. 3e: Dunn's post hoc pairwise comparison test in different age groups across 3 waves. Supp. Tab. 4a: The table presents the results of the independent‐samples Mann‐Whitney U Test, used to compare the distributions of Ct Values in Males and Females. Supp. Tab. 4b: The table summarizes the mean and median COVID‐19 RT‐PCR Ct values in Males and Females. Supp. Tab. 4c: The table presents the results of the [file IID3-14-e70304-s001.docx]

**Supplementary Tables:**

| **Supp. Tab. 1a** Timeline of the three major COVID-19 waves used, showing their start and end months. | |
| --- | --- |
| **COVID-19 waves** | **Months** |
| 1^st^ wave | March 2020 – January 2021 |
| 2^nd^ wave | February 2021 – November 2021 |
| 3^rd^ wave | December 2021 – March 2022 |

| **Supp. Tab. 1b.** The table presents the results of the independent-samples Kruskal-Wallis Test, used to compare the distributions of Ct Values across 3 waves. | | | | | | |
| --- | --- | --- | --- | --- | --- | --- |
| **Null Hypothesis** | **Test** | **Total N** | **Test Statistic** | **Degree Of Freedom** | **Sig. (2-sided test)** | **Decision** |
| The distribution of Ct Value is the same 3 Waves. | Independent-Samples Kruskal-Wallis Test | 52613 | 657.165 | 2 | <.0001 | Reject the null hypothesis. |

| **Supp. Tab. 1c.** Dunn’s post hoc pairwise comparison test across 3 waves | | | |
| --- | --- | --- | --- |
| **Comparisons** | **Test Statistic** | **Sig.** | **Adj. Sig.** |
| 1st vs 2nd wave | 5061.277 | <.0001 | <.0001 |
| 1st vs 3rd wave | 4112.519 | 5.18E-12 | 1.55E-11 |
| 2nd vs 3rd wave | 948.759 | 0.097 | 0.29 |

| **Supp. Tab. 1d.** The table summarizes the mean and median COVID-19 RT-PCR Ct values across 3 COVID-19 waves. | | |
| --- | --- | --- |
| **COVID-19 Waves** | **Mean** | **Median** |
| **1st wave** | 25.22095 | 25 |
| **2nd wave** | 23.09136 | 23 |
| **3rd wave** | 23.43693 | 23.79 |

| **Supp. Tab. 2a** Detailed timeline of each COVID-19 wave used, highlighting pre-peak, peak, and post-peak periods. | |
| --- | --- |
| **COVID-19 wave timelines** | **Months** |
| 1^st^ wave before the peak | March 2020 – May 2020 |
| 1^st^ wave at the peak | September 2020 |
| 1^st^ wave after the peak | December 2020 – January 2021 |
| 2^nd^ wave before the peak | February 2021 |
| 2^nd^ wave at the peak | May 2021 |
| 2^nd^ wave after the peak | June 2021 – November 2021 |
| 3^rd^ wave before the peak | December 2021 |
| 3^rd^ wave at the peak | January 2022 |
| 3^rd^ wave after the peak | March 2022 |

| **Supp. Tab. 2b.** The table presents the results of the independent-samples Kruskal-Wallis Test, used to compare the distributions of Ct Values across different timelines. | | | | | | |
| --- | --- | --- | --- | --- | --- | --- |
| **Null Hypothesis** | **Test** | **Total N** | **Test Statistic** | **Degree Of Freedom** | **Sig. (2-sided test)** | **Decision** |
| The distribution of Ct Value is the same across different timeline | Independent-Samples Kruskal-Wallis Test | 39944 | 473.481 | 8 | <.0001 | Reject the null hypothesis. |

| **Supp. Tab. 2c. Dunn’s post hoc pairwise comparison test across different timeline** | | | |
| --- | --- | --- | --- |
| **Comparisons** | **Test Statistic** | **Sig.** | **Adj. Sig.** |
| 3rd wave before the peak vs 2nd wave after the peak | 345.348 | 0.628 | 1 |
| 3rd wave before the peak vs 2nd wave at the peak | 1015.767 | 0.16 | 1 |
| 3rd wave before the peak vs 3rd wave at the peak | 1623.076 | 0.076 | 1 |
| 3rd wave before the peak vs 2nd wave before the peak | 2280.446 | 0.016 | 0.559 |
| 3rd wave before the peak vs 3rd wave after the peak | 2386.148 | 0.45 | 1 |
| 3rd wave before the peak vs1st wave after the peak | 3371.567 | 0.000053 | 0.002 |
| 3rd wave before the peak vs 1st wave at the peak | 4416.701 | 1.96E-08 | 7.07E-07 |
| 3rd wave before the peak vs 1st wave before the peak | 6166.107 | 2.89E-15 | 1.04E-13 |
| 2nd wave after the peak vs 2nd wave at the peak | 670.419 | 0.000012 | 0.000417 |
| 2nd wave after the peak vs 3rd wave at the peak | 1277.728 | 0.029 | 1 |
| 2nd wave after the peak vs 2nd wave before the peak | 1935.098 | 0.002 | 0.07 |
| 2nd wave after the peak vs 3rd wave after the peak | 2040.8 | 0.508 | 1 |
| 2nd wave after the peak vs 1st wave after the peak | 3026.219 | 9.95E-12 | 3.58E-10 |
| 2nd wave after the peak vs 1st wave at the peak | 4071.352 | 0 | 0 |
| 2nd wave after the peak vs 1st wave before the peak | 5820.759 | 0 | 0 |
| 2nd wave at the peak vs 3rd wave at the peak | 607.309 | 0.308 | 1 |
| 2nd wave at the peak vs 2nd wave before the peak | 1264.679 | 0.047 | 1 |
| 2nd wave at the peak vs 3rd wave after the peak | 1370.381 | 0.657 | 1 |
| 2nd wave at the peak vs 1st wave after the peak | 2355.8 | 3.07E-07 | 0.000011 |
| 2nd wave at the peak vs 1st wave at the peak | 3400.934 | 0 | 0 |
| 2nd wave at the peak vs 1st wave before the peak | 5150.34 | 0 | 0 |
| 3rd wave at the peak vs 2nd wave before the peak | 657.37 | 0.439 | 1 |
| 3rd wave at the peak vs 3rd wave after the peak | 763.072 | 0.808 | 1 |
| 3rd wave at the peak vs 1st wave after the peak | 1748.491 | 0.016 | 0.585 |
| 3rd wave at the peak vs 1st wave at the peak | 2793.625 | 0.000032 | 0.001 |
| 3rd wave at the peak vs 1st wave before the peak | 4543.031 | 8.64E-12 | 3.11E-10 |
| 2nd wave before the peak vs 3rd wave after the peak | 105.702 | 0.973 | 1 |
| 2nd wave before the peak vs 1st wave after the peak | 1091.121 | 0.151 | 1 |
| 2nd wave before the peak vs 1st wave at the peak | 2136.255 | 0.003 | 0.091 |
| 2nd wave before the peak vs 1st wave before the peak | 3885.661 | 2.98E-08 | 0.000001 |
| 3rd wave after the peak vs 1st wave after the peak | 985.419 | 0.751 | 1 |
| 3rd wave after the peak vs 1st wave at the peak | 2030.553 | 0.512 | 1 |
| 3rd wave after the peak vs 1st wave before the peak | 3779.959 | 0.222 | 1 |
| 1st wave after the peak vs 1st wave at the peak | 1045.133 | 0.06 | 1 |
| 1st wave after the peak vs 1st wave before the peak | 2794.54 | 3.30E-07 | 0.000012 |
| 1st wave at the peak vs 1st wave before the peak | 1749.407 | 0.000207 | 0.007 |

| **Supp. Tab. 2d.** The table summarizes the mean and median COVID-19 RT-PCR Ct values across different timelines. | | |
| --- | --- | --- |
| **COVID-19 Timeline** | **Mean** | **Median** |
| 1st wave before the peak | 26.17886 | 26.855 |
| 1st wave at the peak | 25.20849 | 25.675 |
| 1st wave after the peak | 24.80289 | 25 |
| 2nd wave before the peak | 24.21315 | 24 |
| 2nd wave at the peak | 23.31856 | 23.56 |
| 2nd wave after the peak | 22.98677 | 23 |
| 3rd wave before the peak | 22.90042 | 23 |
| 3rd wave at the peak | 23.56744 | 24 |
| 3rd wave after the peak | 24.08857 | 24.5 |

| **Supp. Tab. 3a.** The table presents the results of the independent-samples Kruskal-Wallis Test, used to compare the distributions of Ct Values across different age groups. | | | | | | |
| --- | --- | --- | --- | --- | --- | --- |
| **Null Hypothesis** | **Test** | **Total N** | **Test Statistic** | **Degree Of Freedom** | **Sig. (2-sided test)** | **Decision** |
| The distribution of Ct Value is the same across different Age groups | Independent-Samples Kruskal-Wallis Test | 53266 | 56.815 | 4 | 1.35E-11 | Reject the null hypothesis. |

| **Supp. Tab. 3b.** Dunn’s post hoc pairwise comparison test across different Age Groups | | | |
| --- | --- | --- | --- |
| **Comparisons** | **Test Statistic** | **Sig.** | **Adj. Sig.** |
| Elderly vs Middle Age | 538.078 | 0.008 | 0.084 |
| Elderly vs Adolescence | 1058.446 | 0.000144 | 0.001 |
| Elderly vs Young Adults | 1243.118 | 2.21E-10 | 2.21E-09 |
| Elderly vs Children | 1848.331 | 8.06E-07 | 0.000008 |
| Middle Age vs Adolescence | 520.368 | 0.041 | 0.405 |
| Middle Age vs Young Adults | 705.04 | 0.00001 | 0.000096 |
| Middle Age vs Children | 1310.253 | 0.000241 | 0.002 |
| Adolescence vs Young Adults | 184.672 | 0.456 | 1 |
| Adolescence vs Children | 789.884 | 0.051 | 0.506 |
| Young Adults vs Children | 605.213 | 0.086 | 0.858 |

| **Supp. Tab. 3c.** The table summarizes the mean and median COVID-19 RT-PCR Ct values across different Age groups. | | |
| --- | --- | --- |
| **Age Groups** | **Mean** | **Median** |
| Children | 23.76282 | 24 |
| Adolescence | 23.42281 | 24 |
| Young Adult | 23.52906 | 23.8 |
| Middle Age | 23.25036 | 23 |
| Elderly | 23.01596 | 23 |

| **Supp. Tab. 3d.** The table presents the results of the independent-samples Kruskal-Wallis Test, used to compare the distributions of Ct Values in different age groups across 3 waves. | | | | | | | | |
| --- | --- | --- | --- | --- | --- | --- | --- | --- |
| **Age Group** | **Null Hypothesis** | **Test** | **Total N** | **Test Statistic** | **Degree Of Freedom** | **Sig. (2-sided test)** | **Decision** |  |
| **Children** | The distribution of Ct value is the same in Children across 3 waves | Independent-Samples Kruskal-Wallis Test | 2077 | 23.319 | 2 | 0.000009 | Reject the null hypothesis. |  |
|  |  |  |  |  |  |  |  |  |
| **Adolescence** | The distribution of Ct Value is the same in Adolescence across 3 waves | Independent-Samples Kruskal-Wallis Test | 4631 | 75.23 | 2 | <.0001 | Reject the null hypothesis. |  |
|  |  |  |  |  |  |  |  |  |
| **Young Adults** | The distribution of Ct Value is the same in Young Adults across 3 waves | Independent-Samples Kruskal-Wallis Test | 21022 | 245.507 | 2 | <.0001 | Reject the null hypothesis. |  |
|  |  |  |  |  |  |  |  |  |
| **Middle Age** | The distribution of Ct Value is the same in Middle Age across 3 waves | Independent-Samples Kruskal-Wallis Test | 16261 | 194.882 | 2 | <.0001 | Reject the null hypothesis. |  |
|  |  |  |  |  |  |  |  |  |
| **Elderly** | The distribution of Ct Value is the same in Elderly across 3 waves | Independent-Samples Kruskal-Wallis Test | 8432 | 141.605 | 2 | <.0001 | Reject the null hypothesis. |  |

| **Supp. Tab. 3e.** The table summarizes the mean and median COVID-19 RT-PCR Ct values across different Age groups across 3 waves. | | |
| --- | --- | --- |
| **Comparisons** | **Mean** | **Median** |
| Children_1st_wave | 25.66097 | 26 |
| Children_2nd_wave | 23.49067 | 23.59 |
| Children_3rd_wave | 23.92789 | 23 |
|  |  |  |
| Adolescence_1st_wave | 25.97022 | 26 |
| Adolescence_2nd_wave | 23.13681 | 23 |
| Adolescence_3rd_wave | 23.93382 | 25 |
|  |  |  |
| Young Adults_1st_wave | 25.36414 | 25 |
| Young Adults_2nd_wave | 23.26057 | 23 |
| Young Adults_3rd_wave | 23.91248 | 23.885 |
|  |  |  |
| Middle_Age_1st_wave | 24.94767 | 25 |
| Middle_Age_2nd_wave | 22.98501 | 23 |
| Middle_Age_3rd_wave | 23.1947 | 23.86 |
|  |  |  |
| Elderly_1st_wave | 25.05804 | 25 |
| Elderly_2nd_wave | 22.705 | 23 |
| Elderly_3rd_wave | 22.58651 | 23 |
|  |  |  |

| **Supp. Tab. 3e.** Dunn’s post hoc pairwise comparison test in different age groups across 3 waves | | | | |
| --- | --- | --- | --- | --- |
| **Age Group** | **Comparisons** | **Test Statistic** | **Sig.** | **Adj. Sig.** |
| **Children** | 1st vs 2nd wave | 194.603 | 0.000001 | 0.000004 |
|  | 1st vs 3rd wave | 159.725 | 0.263 | 0.788 |
|  | 2nd vs 3rd wave | 34.877 | 0.801 | 1 |
|  |  |  |  |  |
| **Adolescence** | 1st vs 2nd wave | 573.120 | <.0001 | <.0001 |
|  | 1st vs 3rd wave | 402.601 | 0.009391676 | 0.028175029 |
|  | 2nd vs 3rd wave | 170.518 | 0.233601604 | 0.700804813 |
|  |  |  |  |  |
| **Young Adults** | 1st vs 2nd wave | 1982.515 | <.0001 | <.0001 |
|  | 1st vs 3rd wave | 1366.156 | 0.00025 | 0.000749299 |
|  | 2nd vs 3rd wave | 616.358 | 0.08385939 | 0.251578171 |
|  |  |  |  |  |
| **Middle Age** | 1st vs 2nd wave | 1478.709 | <.0001 | <.0001 |
|  | 1st vs 3rd wave | 1248.883 | 0.000286752 | 0.000860257 |
|  | 2nd vs 3rd wave | 229.825 | 0.489404472 | 1 |
|  |  |  |  |  |
| **Elderly** | 1st vs 2nd wave | 907.335 | <.0001 | <.0001 |
|  | 1st vs 3rd wave | 929.256 | 0.00013546 | 0.000406381 |
|  | 2nd vs 3rd wave | 21.921 | 0.925607303 | 1 |

| **Supp. Tab. 4a** The table presents the results of the independent-samples Mann-Whitney U Test, used to compare the distributions of Ct Values in Males and Females. | | | | | | |
| --- | --- | --- | --- | --- | --- | --- |
| **Null Hypothesis** | **Test** | **Total N** | **Test Statistic** | **Mean Rank** | **Sig.** | **Decision** |
| The distribution of Ct value is the same across Gender. | Independent-Samples Mann-Whitney U Test | 53410 | 359977160.5 | Male (26997.94), Female (26331.25) | 6.97E-07 | Reject the null hypothesis. |

| **Supp. Tab. 4b.** The table summarizes the mean and median COVID-19 RT-PCR Ct values in Males and Females | | |
| --- | --- | --- |
| **Gender** | **Mean** | **Median** |
| **Male** | 23.48176 | 23.78 |
| **Female** | 23.21111 | 23 |

| **Supp. Tab. 4c** The table presents the results of the Independent-Samples Kruskal-Wallis Test, used to compare the distributions of Ct Values in Males and Females across 3 waves. | | | | | | | |
| --- | --- | --- | --- | --- | --- | --- | --- |
| **Gender** | **Null Hypothesis** | **Test** | **Total N** | **Test Statistic** | **Degree Of Freedom** | **Sig.(2-sided test)** | **Decision** |
| Male | The distribution of Ct Value is the same in Male across 3 Waves | Independent-Samples Kruskal-Wallis Test | 29547 | 424.024 | 2 | <.0001 | Reject the null hypothesis. |
|  |  |  |  |  |  |  |  |
| Female | The distribution of Ct Value is the same in Female across 3 Waves | Independent-Samples Kruskal-Wallis Test | 23054 | 226.215 | 2 | <.0001 | Reject the null hypothesis. |

| **Supp. Tab. 4d.** The table summarizes the mean and median COVID-19 RT-PCR Ct values in Males and Females across 3 waves | | |
| --- | --- | --- |
| **Comparisons** | **Mean** | **Median** |
| 1st_wave_Male | 25.35129 | 25.33 |
| 2nd_wave_Male | 23.18075 | 23 |
| 3rd_wave_Male | 23.66329 | 24 |
|  |  |  |
| 1st_wave_Female | 25.00939 | 25 |
| 2nd_wave_Female | 22.97819 | 23 |
| 3rd_wave_Female | 23.15925 | 23 |
|  |  |  |

| **Supp. Tab. 4e.** Dunn’s post hoc pairwise comparison test in Males and Female across 3 waves | | | | |
| --- | --- | --- | --- | --- |
| **Gender** | **Comparisons** | **Test Statistic** | **Sig.** | **Adj. Sig.** |
| **Male** | 1st vs 2nd wave | 2939.082 | <.0001 | <.0001 |
|  | 1st vs 3rd wave | 2219.366 | 7.60571E-07 | 2.28171E-06 |
|  | 2nd vs 3rd wave | 719.716 | 0.09589447 | 0.287683411 |
|  |  |  |  |  |
| **Female** | 1st vs 2nd wave | 2070.849 | <.0001 | <.0001 |
|  | 1st vs 3rd wave | 1826.206 | 3.29E-06 | 9.88E-06 |
|  | 2nd vs 3rd wave | 244.643 | 0.512580092 | 1 |

| **Supp. Tab. 5.** Linear regression analysis depicting the relationship between COVID-19 RT-PCR Ct values and various epidemiological indicators. | | | | | |
| --- | --- | --- | --- | --- | --- |
| **Comparison** | **R^2^** | **R** | **p-value** | **Pearson Correlation** | **Sig.(2-tailed)** |
| Ct value - New cases smoothed | 0.121565 | 0.348661 | 0.029606 | -.349 | 0.029606 |
| Ct value - New cases smoothed per million | 0.121565 | 0.348661 | 0.029606 | -.349 | 0.029606 |
| Ct value - New deaths | 0.097167 | 0.311717 | 0.053388 | -0.31172 | 0.053388 |
| Ct value - New deaths per million | 0.097162 | 0.311708 | 0.053394 | -0.31171 | 0.053394 |
| Ct value - New deaths smoothed per million | 0.151704 | 0.389492 | 0.01425 | -.389 | 0.01425 |
| Ct value - New deaths smoothed | 0.151704 | 0.389492 | 0.01425 | -.389 | 0.01425 |
